# Editorial: Personalized care in cardiac arrhythmias: the role of digital platforms in cardiac arrhythmia management

**DOI:** 10.3389/fcvm.2025.1652279

**Published:** 2025-07-23

**Authors:** Constantinos Bakogiannis, Sotirios Nedios, Nikolaos Papageorgiou, Vassilios Vassilikos

**Affiliations:** ^1^ Third Department of Cardiology, Aristotle University of Thessaloniki, Thessaloniki, Greece; ^2^ Department of Electrophysiology, Heart Center, University of Leipzig, Leipzig, Germany; ^3^ Department of Electrophysiology, Barts Heart Centre, St. Bartholomew’s Hospital, London, United Kingdom

**Keywords:** digital health, personalized medicine, arrhythmias, artificial intelligence, digital health platforms

**Editorial on the Research Topic**
Personalized care in cardiac arrhythmias: the role of digital platforms in cardiac arrhythmia management

Cardiac arrhythmias are complex disorders that affect millions globally. Precise prediction, early diagnosis, and person-centered therapeutic management is of key importance in the era of digital health evolution. Digital technologies offer opportunities for precision medicine, enabling real-time monitoring of patients with arrythmias, while also enhancing patient safety during interventional and non-interventional therapeutic managing. This Research Topic brings together original and diverse contributions that highlight existing gaps and needs in arrhythmia management, also demonstrating various perspectives on how digital tools could facilitate innovative pathways and management strategies to overcome these challenges.

## Remote expertise implementation

Curcio et al. demonstrated the integration of telemedicine into device implantation through real-time remote support for ICD/CRT-D procedures. Replacing on-site clinical bioengineers with internet-based remote guidance, can achieve 100% procedural success and significantly reduce operative times. This highlights telemedicine's potential in overcoming geographical and resource barriers.

## Precision guidance

Liu et al. compared the safety and effectiveness of high-power short-duration (HPSD) to low-power long-duration (LPLD) ablation strategies when used with electroanatomic mapping (EAM) and intracardiac echocardiography (ICE)-guided zero-fluoroscopy procedures. They find that HPSD significantly shortens procedure and ablation times while maintaining comparable efficacy and safety to LPLD. Thus, the use of zero-fluoroscopy workflow with EAM and ICE is safe and feasible for ablation procedures of atrial fibrillation (AF).

Gagyi et al. studied the hypothesis that charge density mapping (CDM)-guided catheter ablation (CA) of post-surgical atrial tachycardias (AT) is safe, feasible, and more effective compared to 3D mapping-based CA. The results of this study demonstrated that CDM is safe and feasible in post-surgical ATs and might be considered superior over conventional 3D mapping as it resulted in significantly lower recurrence rates (10% vs. 46.7% at one-year follow-up). Therefore, advanced mapping systems like CDM could enable personalized ablation strategies for challenging substrates.

## Individualized therapy

Zhou et al.'s network meta-analysis identified optimal exercise types for improving health-related quality of life (HRQoL) in AF patients. Aerobic exercise and Cardiac rehabilitation (CR), which is a combination of aerobic, resistance, and flexibility exercises with patient education and counseling to reduce cardiovascular risk effectively improved the physical and mental component of HRQoL. High-intensity interval training (HIIT) was considered as more beneficial with regard to the mental component of HRQoL. This work provides evidence-based guidance for tailoring exercise programs to individual AF patient profiles. This could be useful to guide exercise intervention via digital health platforms that monitor adherence and physiological responses.

Risk stratification for cardiac arrhythmias is of paramount importance. Järvensivu-Koivunen et al., studied the potential role of computer-interpreted ECG (CIE) in sudden cardiac death (SCD) prediction. Machine learning algorithms were used for the analysis of CIE derived by 8,568 patients with acute coronary syndromes who were followed-up for five years. ECG risk features derived by CIE statements, such as QRS duration, QTc, and the presence of PVCs were detected as predictors of long-term SCD risk. Sensitivity of CIE data to correctly identify patients at high SCD risk was low. The high specificity (96.9%) and negative predictive value (97.3%) position this tool as a scalable “rule-out” mechanism for low-risk populations.

## Complication prevention

A case report by Zhang et al. describes a case of cerebral infarction following the removal of a temporary transvenous pacemaker lead due to interventricular septal perforation. This rare case underscores the need for careful periprocedural management, including the focused use of imaging tools, the use of effective anticoagulation strategies, to prevent such complications.

Collectively, the articles included in this Research Topic highlight four pillars of digital-enabled personalized arrhythmia care: (1) Remote expertise implementation; (2) Precision guidance; (3) Individualized therapy or management selection, and (4) Complication prevention. Challenges remain, including interoperability of digital health tools that limit wider implementation and use of digital platforms, as well as validation in diverse populations, and ensuring equitable access. Future work must prioritize the development of digital mature health ecosystems that allow the collection of high-quality data, the integration of AI-enabled prediction and management tools, the remote monitoring of patients with arrhythmias, the embedding of patient-centered data towards the creation of effective person-centered care workflows.

